# Cina in Spasmodic Cough

**Published:** 1885-03

**Authors:** E. M. Hale


### CINA IN SPASMODIC COUGH.

I do not report this case either as illustrating the value of a high potency, or the power of the medicine, but to point out some errors which have been a stumbling-block to our school, retarding its progress more than we have supposed.

A child about four years of age had been under the treatment of Professor D——, of this city, an allopath, for nearly four months for the following symptoms, which have not been ameliorated:

(*a*) A cough day and night, worse at night, of a peculiarly distressing character. The paroxysms occurred every two or three hours, during which the child would become exhausted, have difficulty in regaining the breath, and often end in a "spasm." (This spasm was described by the nurse as a rigidity of the whole body, or a convulsive throwing of the body backward so sudden as to force the child off her lap.)

(*b*) Ravenous appetite, never satisfied, eats heartily, has hard, disturbed abdomen, picks constantly at its nose, rubs its nose in its sleep, has stools of mucus and undigested food.

Of course, any homœopathician would select *Cina.* The characteristic symptoms all correspond. I selected the two hundredth dilution, not from any particular belief in the superior efficiency of that potency, for *Cina* will cure those symptoms in any potency, even in drop-doses of the mother tincture, as I have often verified.

Two drops of the two hundredth were dropped into a half-ounce vial of dilute alcohol. The vial was a new one, just from the manufactory, dingy, dirty, and of green glass. It was simply rinsed in cold water—no elaborate boiling, cleaning, or drying.

Ten drops of this preparation were ordered three times a day, given on a lump of sugar. (The child had been without medicine for a week and was growing worse, and the recovery could not have been a coincidence.)

Three days of this medicine relieved the cough so much that only a few light paroxysms occurred in the twenty-four hours, and in two weeks the child was quite well.

The point I wish to illustrate is this: We have been taught that a most scrupulous cleansing of vials, a most immaculate purity of water, alcohol, or pellets, was absolutely necessary to the proper administration and preparation of the high potencies. *I do not believe such extreme precautions are of the slightest consequence. If the curative power in a drug is a force, as we all believe it to be, such force must be of a fixed, immutable, and unchangeable character.* It can only be influenced by peculiar chemical substances, or dynamic agents for which it may have an affinity or an antagonism. Simple uncleanliness cannot influence it, nor can the majority of crude medicinal agents. A drop of *Cina*$^{200}$ will, I believe, act as well when mixed with a tumbler of muddy water, milk, tea, or almost any vehicle which may be named. I have seen one drop of Belladonna$^{30}$ act finely when given in a tumbler of Chicago hydrant water, when it is actually putrid with the foul emanations of the river (that was before the days of the lake tunnel). I know that *Calcarea*$^{30}$ acts finely when given in the milk which a child draws through a nursing bottle. How is it that our high potencies act curatively when given in putrid states of the system, as in typhus, when administered by the mouth? One cannot well imagine a filthier place than the mouth and stomach of a man sick with typhus.

It was one of the saddest mistakes of our school that the odors of flowers, the eating and drinking of certain articles of food and beverages, were forbidden our patients. The curative force cannot be influenced by such agencies. It is as immutable and indestructible in the thirtieth as in the first decimal dilution. Let us divest ourselves of this fear of uncleanliness in the vehicles in which we use our attenuated medicines, and act as if they represented the fixed forces which we believe them to possess. By so doing we shall remove the chief obstacle to their general employment and elevate our doctrine of the dynamic power of drugs to the dignity of a scientific fact.—E. M. HALE *in A. J. M. M.*

---

A NUT FOR DR. DUDGEON TO CRACK.—Dr. Dudgeon asserts that Hahnemann advised Camphor in cholera, because it destroyed the supposed germs. If so, why did he advise Camphor only in the first stage, and why did he advise Cuprum, Veratrum, etc., in the later stages in the 30th potency? Do the supposed germs cease to exist in the later stages, or will the 30th potency act as a germicide? And if the cholera microbes, like the diphtheritic bacteria, turn out to be only fibrin, what becomes of Dr. Dudgeon's ingenious attempt to force Hahnemann's Homœopathy into a "pathological strait-waiscoat"? E. W. BERRIDGE, M. D.

